# Analysis of the early cellular and humoral responses of *Galleria mellonella* larvae to infection by *Candida albicans*

**DOI:** 10.1080/21505594.2017.1370174

**Published:** 2017-09-21

**Authors:** Gerard Sheehan, Kevin Kavanagh

**Affiliations:** Department of Biology, Maynooth University, Maynooth, Co. Kildare, Ireland

**Keywords:** candida, galleria, immunity, infection, *in vivo* screening, Mini-model

## Abstract

*Galleria mellonella* larvae were administered an inoculum of *Candida albicans* and the response to infection over 24 hours was monitored. The yeast cell density in infected larvae declined initially but replication commenced six hours post-infection. The hemocyte density decreased from 5.2 × 10^6^/ml to 2.5 × 10^6^/ml at 2 hours but increased to 4.2 × 106 at 6 hours and decreased subsequently. Administration of β – glucan to larvae also caused a fluctuation in hemocyte density (5.1 ± 0.22 × 10^6^/ml (0 hour) to 6.25 ± 0.25 × 106/ml (6 hour) (p < 0.05) to 5 ± 2.7 × 106 (24 hour)) and the population showed an increase in the density of small, granular cells at 24 hours (p < 0.05). Hemocytes from larvae inoculated with β – glucan for 6 or 24 hours showed faster killing of *C. albicans* cells (53 ± 4.1% (p < 0.01), 64 ± 3.7%, (p < 0.01), respectively) than hemocytes from control larvae (24 ± 11%) at 60 min. Proteomic analysis indicated increased abundance of immune related proteins cecropin-A (5 fold) and prophenoloxidase-activating proteinase-1 (5 fold) 6 hours post infection but by 24 hours there was elevated abundance of muscle (tropomyosin 2 (141 fold), calponin (66 fold), troponin I (62 fold)) and proteins indicative of cellular stress (glutathione-S-transferase-like protein (114 fold)), fungal dissemination (muscle protein 20-like protein (174 fold)) and tissue breakdown (mitochondrial cytochrome c (10 fold)). Proteins decreased in abundance at 24 hour included β – 1,3 – glucan recognition protein precursor (29 fold) and prophenoloxidase subunit 2 (25 fold).

## Introduction

Due to the structural and functional similarities that exist between the insect immune system and the innate immune response of mammals, insects have been widely used to study the virulence of pathogens such as *Aspergillus fumigatus*, *Pseudomonas aeruginosa* and *Candida albicans* and generate results that correlate with those that may be obtained using mammals.^1-5^ A wide range of insect species are now employed to assess the virulence of fungal and bacterial pathogens or to evaluate the *in vivo* toxicity and efficacy of antimicrobial drugs.^6^^,^^7^ Larvae of the greater wax moth, *Galleria mellonella* are a popular choice for these types of assays due to their ease of inoculation, cost effectiveness and the lack of legal and ethical restraints to their use. A number of end points may be used to evaluate the response of larvae to infection including mortality, extent of melanisation, alteration in hemocyte population or function, and alteration in proteomic profile. A correlation between alterations in the hemocyte density in larvae and the relative virulence of fungal pathogens has been established.^8^

The innate immune system of mammals is evolutionarily ancient and displays many cellular and humoral similarities to the insect immune response. The cellular immune response of insects is mediated by hemocytes which display the ability to phagocytose and kill pathogens. Insect hemocytes possess a functional NADPH oxidase complex capable of generating superoxide and incorporating proteins with homology to p40^phox^, p47^phox^, p67^phox^ and gp91^phox^ of mammalian neutrophils.^9^ Mammalian neutrophils and insect hemocytes display similar responses to the *A. fumigatus* toxin fumagillin, nocodazole (inhibitor of tubulin formation) and cytochalasin B (weakens actin formation).^10-12^ Neutrophils and hemocytes engage in lectin-mediated phagocytosis, and share homologous receptors on their cell surfaces for pathogen recognition, cellular communication and migration. Hemocyte Toll and Immune Deficiency pathways activate NF-κB-like mediated anti-microbial peptide (AMP) production, with adult *Drosophila* Toll/ Dorsal signaling choreographing the insect anti-fungal immune response.^13^ Insects produce a range of AMPs which are synthesized by the insect fat body and bioactivated at sites of wounding in response to non-self detection. *G. mellonella* larvae produce the AMPs gallerimycin, defensin, moricin, gloverin and cecropin-like peptides.^14^ The cationic amphipathic alpha-helical cecropin-A induces *C. albicans* apoptosis, whereas moricins are highly basic and display broad spectrum antibacterial activity, defensins are evolutionary conserved cysteine-rich peptides essential in anti-fungal invertebrate and vertebrate immunity.^15^^,^^16^ Furthermore, the antimicrobial protein lysozyme, from *G. mellonella* at physiological concentrations induces *C. albicans* apoptosis in a potassium channel dependent manner.^17^

While the use of *G. mellonella* larvae has greatly facilitated research, little attention has been directed to the study of the early stages of the pathogen – host interaction. The characterization of the early stages of infection may facilitate the use of *G. mellonella* larvae to study the initial pathogen – mammalian host interactions. Pre-exposure of *G. mellonella* larvae to a sub-lethal infection by *C. albicans* primes the immune response and larvae show reduced susceptibility to subsequent lethal infection.^8^ We previously established the role of thermal and physical stresses in priming the immune response to infection and this was mediated by an increase in the hemocyte density and in the expression of AMPs.^18^^,^^19^ *G. mellonella* larvae are capable of discriminating between the extent of infection and mounting dose dependent cellular and humoral responses to β - glucan and this effect was mediated by alterations in the density of circulating hemocytes and in the abundance of a range of immune related proteins.^20^ Larvae infected with different doses of *A. fumigatus* conidia displayed differential activation of cellular and humoral immune responses.^21^ A similar immune priming effect was observed in *G. mellonella* larvae when administered the anti-fungal drug caspofungin, which increased the resistance of the larvae to fungal and bacterial infection.^22^ It was also demonstrated that prolonged pre-incubation of *G. mellonella* larvae resulted in reduced resistance to infection by bacterial and fungal pathogens as a result of lower levels of hemocytes and reduced AMP expression.^23^

Analysis of the early stages of this pathogen–host interaction may highlight additional end points that could be used to assess and quantify the larval immune response. In addition characterizing the early stages of infection may allow the development *of G. mellonella* larvae as a model to study initial stages of pathogen – mammal infection.

## Results

### Response of *G. mellonella* to *C. albicans* infection

**Figure 1.**
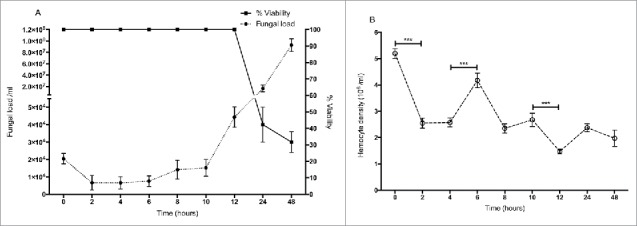
(A) Survival of larvae and fluctuations in fungal cell density in *C. albicans* infected larvae. (B) Changes in hemocyte density in response to inoculation with *C. albicans*. (***: p < 0.001). Square: larval survival, closed circle: fungal load, open circle: hemocyte density. All values are the mean ± SD of three independent replicates.

Larvae were inoculated by intra-hemocoel injection with 5 × 10^5^ yeast cells as described and incubated at 30 °C for 48 hour. Larvae demonstrated no mortality from 0 – 12 hour but by 24 hour mortality was 60 ± 10% and 70 ± 6% at 48 hour (Fig. 1A) The yeast cell density in infected larvae decreased from 2.1 ± 0.35 × 10^4^/ml at time 0 to 0.66 ± 0.05 × 10^4^/ml (p < 0.01) at 2 hour. Yeast cell density remained stable until 6 hour (0.77 ± 0.15 × 10^4^/ml) but increased to 4.4 ± 0.06 × 10^4^/ml at 12 hour. By 24 hour the yeast cell density had reached 1.68 ± 0.15 × 10^7^/ml and at 48 hour had reached 9.38 ± 1.7 × 10^7^/ml (Fig. 1A). The density of circulating hemocytes initially decreased from 5.2 ± 0.2 × 10^6^ to 2.55 ± 0.2 × 10^6^, (p < 0.001) at 2 hour but by 6 hour it had reached 4.2 ± 0.3 × 10^6^ (p < 0.001). By 12 hour the hemocyte density had declined to 1.47 ± 0.1 × 10^6^ (p < 0.001) (Fig. 1B).

### Cellular immune response to yeast **β** – (1, 3) glucan

**Figure 2.**
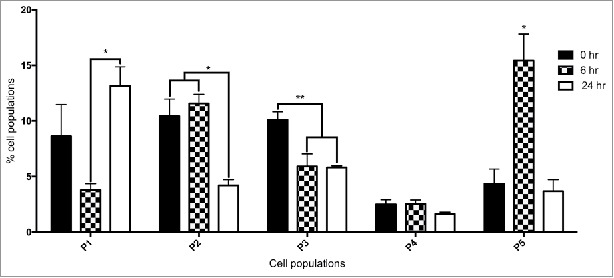
FACS analysis of hemocyte sub-populations. Fluctuations in hemocyte sub-populations in larvae administered β – glucan at 0 hour, 6 hour and 24 hour. Hemocyte sub-populations were measured based on size and granularity as described (*: p < 0.05, **: p < 0.01). All values are the mean ± SD of 3 independent determinations.

In order to characterize the changes in the hemocyte population by flow cytometry in larvae, *G. mellonella* larvae were administered β – (1, 3) glucan, a component of the yeast cell wall for 0, 6 and 24 hours. β - glucan was employed as it was previously demonstrated to induce an immune response in *G. mellonella* larvae and enabled the use of the FACS facility to differentiate hemocytes without the risk of yeast contamination of the instrument.^24^ Larvae administered β – glucan for 6 or 24 hours had a hemocyte density of 6.25 ± 1.7 × 10^6^ (p < 0.05) and 5 ± 2.7 × 10^6^, respectively. PBS injected larvae, herein referred to as control larvae showed a hemocyte density of 5.1 ± 1.6 × 10^6^. FACS analysis was employed to establish if there was a change in the relative proportion of each hemocyte sub-population in naïve larvae and in those larvae administrated β – glucan for 6 and 24 hours. Hemocyte populations were differentiated on the basis of size and granularity and at least 5 distinct sub-populations,^23^ labeled P1 to P5, were identified, (Fig. 2). Larvae administered β – glucan for 24 hours showed a significant increase (p < 0.05) in the relative abundance of P1 hemocytes (small, granular cells) as compared to control larvae. The total percentage of the P1 sub-population of hemocytes in 6 hours β – glucan challenged larvae was 3.78 ± 0.56% and 13.15 ± 1.7% in larvae administered β – glucan for 24 hours. There was a significant increase in P5 hemocytes (very large, non granular cells) at 6 hour β – glucan exposure. P2 and P3 (medium sized, granular cells) were both significantly decreased at 24 hours compared to naïve larvae.

**Figure 3.**
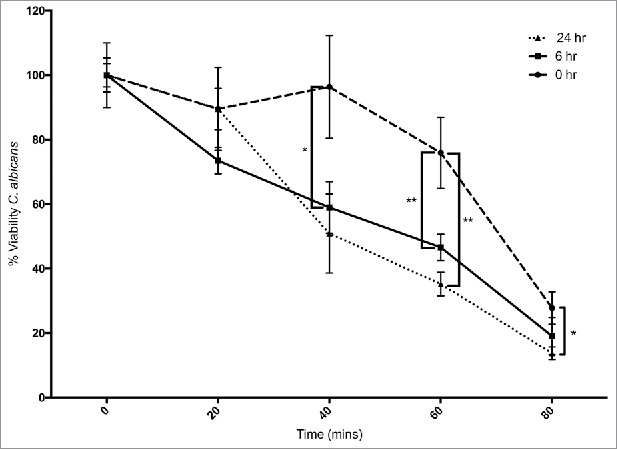
*Ex vivo* fungicidal activity of hemocytes from *G. mellonella* larvae administrated 20 μg β-1,3-glucan/ larva (*: p < 0.05, **: p < 0.01). Circle: 0 hour, square: 6 hour, triangle: 24 hour treated larvae. All values are the mean ± SD of 3 independent replicates.

Hemocytes were extracted from β – glucan administered larvae and their ability to kill *C. albicans* cells was assessed as described (Fig. 3). Hemocytes from control larvae killed 24 ± 11% of the yeast cells after 60 min, while hemocytes from larvae administered β – glucan for 6 and 24 hours killed 53 ± 4% and 65 ± 3.6%, (p < 0.01) after 60 min, respectively. At 80 minutes hemocytes from control larvae killed 72 ± 5% yeast cells while those from larvae administered β – glucan for 6 or 24 hours killed 81 ± 5.7% or 86 ± 1.9% (p < 0.05), respectively.

### Analysis of Proteomic response of *G. mellonella* larvae to *Candida* infection

**Figure 4.**
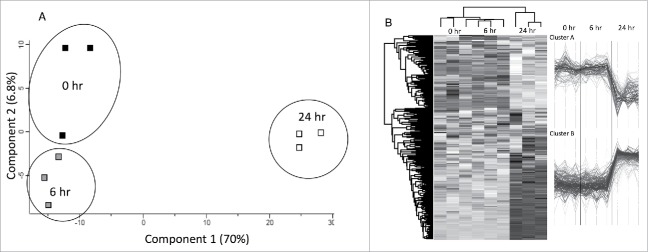
Shotgun quantitative proteomic analysis of *G. mellonella* infected with *C. albicans*. (A) Principal component analysis (PCA) of *G. mellonella* infected with *C. albicans* at 0, 6 and 24 hours with a clear distinction between each time point. (B) Two-way unsupervised hierarchical clustering of the median protein expression values of all statistically significant differentially abundant proteins. Hierarchical clustering (columns) identified 3 distinct clusters comprising the three replicates from their original sample groups.

Label free quantitative proteomic analysis was conducted on the *G. mellonella* cell free hemolymph proteome after exposure to *C. albicans* for 0, 6 and 24 hours. In total, 3167 peptides were identified representing 586 proteins with two or more peptides and 21, 125 and 113 (6 hr v 0 hr, 24 hr v 0 hr, 24 hr v 6 hr, respectively) proteins were determined to be differentially abundant (ANOVA, p < 0.05) with a fold change of > 2. A total of 55 proteins were deemed exclusive (i.e. with LFQ intensities present in all three replicates of one treatment and absent in all three replicates of the other two treatments). These proteins were also used in statistical analysis of the total differentially expressed group following imputation of the zero values as described. After data imputation these proteins were included in subsequent statistical analysis. A principal component analysis (PCA) performed on all filtered proteins distinguished the 0, 6 and 24 hour *C. albicans* treated samples indicating a clear difference between each proteome (Fig. 4A). Hierarchical clustering of z-score normalized intensity values for all significantly differentially abundant proteins (n = 259) produced the three replicates of each sample group (Fig. 4b). Furthermore, 2 major protein clusters were identified: 0 hour and 6 hour abundant proteins (Cluster A) and 24 hour abundant proteins (Cluster B).

**Figure 5.**
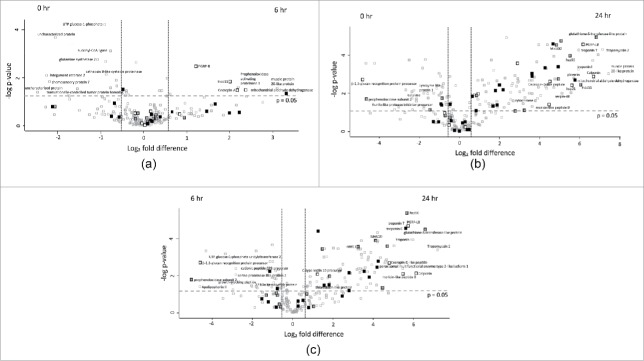
Volcano plots of all identified proteins based on relative abundance differences between *G. mellonella* larvae treated at 0, 6 or 24 hour. Volcano plots showing the distribution of quantified proteins according to p value (−log10 p-value) and fold change (log2 mean LFQ intensity difference). Proteins above the line are considered statistically significant (p value < 0.05) and those to the right and left of the vertical lines indicate relative fold changes ± 1.5. The top 20 differentially abundant proteins are annotated and all proteins associated with the stress response (grey), immune response (white) and oxidoreductase activity (black) are highlighted for (5A) 6 hour and 0 hour, (5B) 24 hour and 0 hour and (5C) 24 hour and 6 hour *C. albicans* treated larvae.

Proteins increased in relative abundance in the larvae challenged with *C. albicans* for 6 hours compared to the control were mitochondrial aldehyde dehydrogenase (10 fold increased), prophenoloxidase-activating proteinase-1 (5 fold increased), Cecropin-A (4.5 fold increased), Hdd11 (4 fold increased) and peptidoglycan recognition – like protein B (2.3 fold increased) (Fig. 5A). Proteins decreased in relative abundance in the larvae challenged with *C. albicans* for 6 hour compared to the control were uncharacterized protein (5.6 fold decreased), integument esterase 2 precursor (4.8 fold decreased), chemosensory protein 7 precursor (4.6 fold decreased) and translationally – controlled tumor protein homolog (2.9 fold decreased), (Fig. 5A).

Proteins increased in relative abundance in larvae inoculated with *C. albicans* for 24 hours compared to the control larvae were muscle protein 20-like protein (174 fold increased), tropomyosin 2 (141 fold increased), glutathione-S-transferase-like protein (114 fold increased), peptidoglycan recognition protein – LB (72 fold increased), mitochondrial aldehyde dehydrogenase (54 fold increased) gloverin (52 fold increased), Hdd11 (49 fold increased), serpin-4B (35 fold increased), Mn superoxide dismutase (30 fold increased), cecropin-D (23 fold increased) and moricin-like peptide B (21 fold increased). Proteins decreased in relative abundance in larvae inoculated with *C. albicans* for the 24 hours were β – 1,3 – glucan recognition protein precursor (28.6 fold decreased), prophenoloxidase subunit 2 (25 fold decreased), catalase (5 fold decreased), Kunitz-like protease inhibitor precursor (5 fold decreased), lysozyme-like protein 1 (5 fold decreased) and putative Gram negative bacteria – binding protein precursor (4.5 fold decreased) (Fig. 5b).

Proteins increased in relative abundance in the 24 hour versus the 6 hour *C. albicans* exposed larvae were tropomyosin 2 (146 fold increased), glutathione-S-transferase-like protein (96 fold increased), troponin I (63 fold increased), peptidoglycan recognition protein – LB (53 fold increased), heat shock protein 90 (51 fold increased), moricin-like peptide B (44 fold increased), cecropin-D-like peptide (28 fold increased), gloverin (25 fold increased) and mitochondrial Mn superoxide dismutase (17 fold increased). Proteins decreased in relative abundance in the 24 hour infected larvae compared to the 6 hour *C. albicans* exposed larvae were prophenoloxidase subunit 2 (32 fold decreased), apolipophorin II (24 fold decreased), β – 1,3 – glucan recognition protein precursor (24 fold decreased), serine proteinase-like protein 2 (7 fold decreased), Kunitz-like protease inhibitor precursor (5 fold decreased) and 27 kDa hemolymph protein (4 fold decreased) (Fig. 5C).

## Discussion

Larvae of *G. mellonella* are now widely used for assessing the virulence of microbial pathogens and for evaluating *in vivo* activity of antimicrobial drugs. *G. mellonella* larvae have also been used to assess the *in vivo* toxicity of food preservatives and a variety of novel antimicrobial drugs and the results show a strong correlation to those obtained using mammals.^22^^,^^23^^,^^25-27^ The similarities between the immune responses of insects and the innate immune response of mammals can be further exploited through using insects as models to study disease development and progression.^1^^,^^27^ *G. mellonella* larvae infected with *Listeria* show neural pathologies similar to those in mammals thus opening the possibility of examining neural disease and repair mechanisms in insects.^28^ This finding could enable the use of *G. mellonella* larvae for studying brain development and for rapidly evaluating the efficacy of novel drugs designed to treat neural diseases or malfunction.

The results presented here describe the development of *C. albicans* in *G. mellonella* larvae over 24 hours and detail the initial cellular and humoral immune responses that the insect employs to limit fungal development. Larvae administered *C. albicans* showed 60 ± 10% mortality at 24 hours and 70 ± 6% at 48 hours. Following inoculation with *C. albicans*, yeast cell density decreased between 0 and 2 hours which may be associated with nodulation by hemocytes at the focal point of infection.^1^^,^^29^^,^^30^ The hemocyte density showed an increase at 6 hours possibly due to the release of sessile hemocytes normally attached to the fat body and inner surface of the hemocoel of larvae.^8^^,^^31^ It has been demonstrated that thermal or physical stress can lead to an elevated hemocyte density and this can be associated with decreased susceptibility to infection.^19^^,^^23^ Yeast cell proliferation in larvae did not commence until 8 hours post infection and then rose to 1.68 ± 0.15 × 10^7^ /ml at 24 hours and 9.38 ± 1.7 × 10^7^/ml at 48 hours.

In order to characterize the changes in the hemocyte population in larvae, larvae were administered β - glucan which also produced a rise in hemocyte density at 6 hours. FACS analysis demonstrated an increase in the relative abundance of large, non granular cells (P5) at 6 hours. At 24 hours post β - glucan administration there was an increase in the relative abundance of small, granular cells (P1). Hemocytes from larvae administered β - glucan for 6 or 24 hours demonstrated a superior ability to kill *C. albicans* cells thus indicating a higher proportion of microbicidal active cells in the respective populations.

Semi-quantitative shotgun proteomics was employed to analyse the response of *G. mellonella* larvae to *C. albicans* infection at 6 and 24 hours. Six hours following *C. albicans* infection there was an increase in the abundance of immune proteins. These are constitutively expressed and augmentable upon microbial detection, for example prophenoloxidase-activating proteinase-1 (5 fold increased) contributes to insect immunity by converting prophenoloxidase to phenoloxidase which participates in melanotic encapsulation, wound healing, and cuticle sclerotization.^32^ Cecropin-A (4.5 fold increased) is a 37 amino acid antimicrobial peptide first isolated from *H. cecropia*. It demonstrates antibacterial activity against multidrug resistant *A. baumanii* and *P. aeruginosa*, induces *C. albicans* apoptosis and recently has been shown to have immunomodulary effects on macrophages.^15^^,^^33^ Immune related Hdd1 (4 fold increased) was found up-regulated in *Hyphantria cunea* 2 hours following inoculation of *E. coli*.^34^

Muscle proteins (muscle protein 20-like protein, tropomyosin 2, calponin, troponin I, troponin T, troponin C, beta actin) were increased from 30-fold to 175-fold in hemolymph after 24 hours and may be associated with tissue disruption due to fungal proliferation and hyphal formation. Antimicrobial peptides and immune proteins (peptidoglycan recognition protein – LB, gloverin, Hdd11, cecropin-D-like peptide, moricin-like peptide B, hemolin) were increased in abundance in response to *Candida* infection at 24 hours. Gloverins are glycine rich, heat stable antibacterial polypeptides believed to bind LPS and possibly components of the fungal cell wall. It was previously demonstrated that *E. coli* but not yeast or saline injection induced gloverin expression in *Bombyx mori*.^35^ Moricins are secreted pro-peptides under the control of (NF-κB)/Rel and GATA transcription factors and are activated via proteolysis and increase the permeability of bacterial and fungal membranes. *G. mellonella* moricins are highly active against yeasts and filamentous fungi.^36^ Immunoglobulin superfamily member hemolin was induced by *Candida* challenge and has been shown to act as a pattern recognition receptor in insects.^23^^,^^37^

Cellular stress proteins (glutathione-S-transferase-like protein, mitochondrial aldehyde dehydrogenase, heat shock protein hsp21.4, heat shock protein 90, putative mitochondrial Mn superoxide dismutase) were increased from 4-fold to 114-fold in hemolymph at 24 hours and function to protect cells from geno- and proteotoxicity. Mitochondrial aldehyde dehydrogenase is important to protection against nitrate induced oxidative stress in *G. mellonella*.^25^ Immune related proteins (e.g. β – 1,3 – glucan recognition protein precursor (29 fold) and lysozyme-like protein 1 (5 fold)) were decreased in abundance at 24 hours, possible due binding of yeast cells and resulting in decreased abundance in hemolymph. Proteomic profiling of RAW 264.7 macrophages exposed to *C. albicans* demonstrated increased abundance of tropomyosin, β-actin, elongation factor 2, triosephosphate isomerase, heat shock protein 71 kDa, annexin A1 and glyceraldehyde-3-phosphate dehydrogenase which are indicative of oxidative stress, immune response, unfolded protein response and apoptosis.^38^^,^^39^

The response of *G. mellonella* larvae to infection by *C. albicans* can be divided into two phases. In phase 1, extending from 0 – 6 hours, there is a decrease in fungal load per larva correlating with an increase in the hemocyte density and the increased abundance of antimicrobial peptides and immune proteins. In phase 2 (6 – 24 hours) yeast cell proliferation occurs, the hemocyte population declines, larval death occurs and proteomic analysis reveals increased abundance of proteins associated with tissue degradation. In murine invasive candidiasis the kidney is the principle site of dissemination which launches an acute phase response consisting of complement activation, cellular recruitment, protease inhibition and opsonization which is analogous to the initial cellular and humoral response of *G. mellonella* to infection and essential for resistance to *C. albicans* infection.^40^^,^^41^ In a fatal mouse model of systemic invasive candidiasis, yeast cells quickly disseminate from blood to distal locations including the kidney. Inoculation results in an early robust peripheral blood neutrophilia which subsequently decreased between day 4 and 7, followed by murine death.^42^ The murine response in the first 24 hours is crucial as the kidney is relatively neutropenic. The current study found a reduction in hemocyte density possibly as a result of local accumulation (nodulation) of hemocytes, followed by an increase in the population of granular cells (P1) at 24 hours. The early stage of detection and action during systemic infection may be non-specific (e.g. AMPs, melanization, nodulation) in order to gauge the extent of fungal burden and allocate immune resources accordingly. In the second stage from 6 to 24 hour, a large scale, specific response (e.g. granular cells, increased AMP) is launched to an already uncontrollable and disseminated fungal infection (e.g. larval death, increased abundance of muscle proteins).

The results presented here characterize the initial cellular and humoral responses of *G. mellonella* larvae to a lethal infection by *C. albicans* and demonstrate parallels with the response of mammals to infection by this yeast. Understanding these interactions in *G. mellonella* may enable the use of larvae to model this process in mammals and help to fine tune therapy for the control of candidiasis.

## Materials and methods

### Larval culture and inoculation

Sixth instar larvae of the greater wax-moth *G. mellonella* (Livefoods Direct Ltd, Sheffield, England), were stored in the dark at 15 °C to prevent pupation. Larvae weighing 0.22 ± 0.03 g were selected and used within two weeks of receipt. Ten healthy larvae per treatment and controls (n = 3) were placed in sterile nine cm Petri dishes lined with Whatman filter paper and containing some wood shavings. Larvae were inoculated with yeast cells or β - glucan through the last left pro-leg into the hemocoel with a Myjector U-100 insulin syringe (Terumo Europe N.V., Belgium). Larvae were acclimatized to 30 °C for 1 hour prior to all experiments and incubated at 30 °C for all studies. All experiments were performed independently on three separate occasions.

### Yeast strain

*Candida albicans* MEN (a kind gift from Dr. D. Kerridge, Cambridge, UK) was cultured in YEPD broth (2% (w/v) glucose, 2% (w/v) bactopeptone (Difco Laboratories), 1% (w/v) yeast extract (Oxoid Ltd., Basingstoke, England)) at 30 **°C** and 200 rpm in an orbital shaker. Stocks were maintained on YEPD agar plates (as above but supplemented with 2% (w/v) agar).

### Glucan solution

β− 1,3 -glucan derived from *Saccharomyces cerevisiae* (Sigma Aldrich Chemical Co., catalogue number G5011-25MG) (molecular weight 5.85 kDa) was dissolved by vigorous vortexing and sonication in PBS at 1 mg/ ml.

### Determination of hemocyte density

An inoculum of 5 × 10^5^ yeast cells/ larvae was achieved by inoculating larvae with 20 μl of a 2.5 × 10^7^ cells/ ml PBS solution.Larvae were inoculated with 20 μg of β− 1,3 -glucan per 20 μl from a 1 mg/ml PBS solution as determined as 20 μg of β− glucan in known to activate a cellular and humoral response.^24^ Hemocyte density was determined by bleeding 5 larvae (two drops hemolymph (40 μl) / larva) into a pre-chilled micro-centrifuge tube to prevent melanization. The collected hemolymph was diluted in PBS supplemented with 0.37% (v/v) mercaptoethanol and cell density was assessed with a hemocytometer and expressed as hemocytes per ml of hemolymph. Experiments were performed on three independent occasions and the means ± S.D. were determined.

### Determination of fungal load in *G. mellonella* larvae

Three larvae inoculated with *C. albicans* were homogenized using a pestel and mortar in 3 ml of sterile PBS. This was serially diluted with PBS, and 100 µl aliquots of the resulting dilutions were plated on YEPD plates containing erythromycin (1 mg/ml) to prevent bacterial overgrowth. These plates were incubated at 30 °C for 48 hours. The fungal load was calculated as the yeast cell density per larva and was based on the number of colonies that grew at specific dilutions.

### FACS analysis of Hemocytes sub-populations in G. mellonella larvae

Larvae were administered β− 1,3 - glucan in order to allow FACS analysis of hemocyte populations to be performed without interference from *C. albicans* cells in the hemolymph.^23^ Hemolymph was extracted from larvae (n = 20) administered 20 μg of β− 1,3 - glucan as described and diluted in ice cold PBS. Hemocytes were enumerated and the density was adjusted to 1 × 10^6^ cells/ml. Hemocytes were washed in 1% BSA/PBS, 1500 x *g* for 5 min at 4 ^o^C and re-suspended in BSA/PBS at a density of 1 × 10^6^ cells/ml. Hemocyte populations were characterized using a FACS BD Accuri™ C6 cytometer and cells were differentiated based on side and forward scatter with a total of 10,000 events measured per sample.

### Determination of fungicidal activity of hemocytes

Larvae were inoculated with 20 μg of β− 1,3 - glucan and incubated for 0, 6 or 24 hours (n = 10). Hemocytes were extracted from larvae and incubated at a density of 1 × 10^6^/ml at 30 ^o^C in PBS in a thermally controlled stirring chamber. Cell free hemolymph opsonized *C*. *albicans* (2 × 10^6^ cells) was added and killing was measured as described previously^9^ by diluting and plating yeast cell suspensions onto YEPD agar plates. Plates were incubated and the resulting number of colonies enumerated. Results were calculated as the mean (± S.D.) from at least three experiments with colony counts performed in triplicate for each sample and expressed as a percentage of the original number at time zero.

### Label Free Quantitative proteomics of larval hemolymph

Label free shotgun semi-quantitative proteomics was conducted on hemocyte-free hemolymph from larvae (n = 5) at 0, 6 and 24 hours post infection with *C. albicans* (5 × 10^5^/ larva). Protein (75 μg) was reduced with dithiothreitol (DTT; 200 mM) (Sigma-Aldrich), alkylated with iodoacetamide (IAA; 1 M) (Sigma-Aldrich) and digested with sequence grade trypsin (Promega, Ireland) at a trypsin:protein ratio of 1:40, overnight at 37 °C. Tryptic peptides were purified for mass spectrometry using C18 spin filters (Medical Supply Company, Ireland) and 1 μg of peptide mix was eluted onto a Q-Exactive (ThermoFisher Scientific, U.S.A) high resolution accurate mass spectrometer connected to a Dionex Ultimate 3000 (RSLCnano) chromatography system. Peptides were separated by an increasing acetonitrile gradient on a Biobasic C18 Picofrit™ column (100 mm length, 75 mm ID), using a 65 min reverse phase gradient at a flow rate of 250 nL /min. All data was acquired with the mass spectrometer operating in automatic data dependent switching mode. A high resolution MS scan (300-2000 Dalton) was performed using the Orbitrap to select the 15 most intense ions prior to MS/MS.

Protein identification from the MS/MS data was performed using the Andromeda search engine in MaxQuant (version 1.2.2.5; http://maxquant.org/) to correlate the data against a 6-frame translation of the EST contigs for *G. mellonella.*^43^*^,^*^44^ The following search parameters were used: first search peptide tolerance of 20 ppm, second search peptide tolerance 4.5 ppm, carbamidomethylation of cysteines was set as a fixed modification, while oxidation of methionines and acetylation of N-terminals were set as variable modifications and a maximum of 2 missed cleavage sites allowed. False Discovery Rates (FDR) were set to 1% for both peptides and proteins and the FDR was estimated following searches against a target-decoy database. Peptides with minimum length of seven amino acid length were considered for identification and proteins were only considered identified when more than one unique peptide for each protein was observed. The MS proteomics data and MaxQuant search output files have been deposited to the ProteomeXchange Consortium^45^ via the PRIDE partner repository with the dataset identifier PXD006879.

Results processing, statistical analyses and graphics generation were conducted using Perseus v. 1.5.5.3. LFQ intensities were log_2_-transformed and ANOVA of significance and t-tests between the hemolymph proteomes of 0, 6 and 24 hours *C. albicans* treated larvae was performed using a p-value of 0.05 and significance was determined using FDR correction (Benjamini-Hochberg). Proteins that had non-existent values (indicative of absence or very low abundance in a sample) were also used in statistical analysis of the total differentially expressed group following imputation of the zero values using a number close to the lowest value of the range of proteins plus or minus the standard deviation. After data imputation these proteins were included in subsequent statistical analysis.

### Statistical analysis

All experiments were performed on three independent occasions and results are expressed as the mean ± S.D. Analysis of changes in hemocyte density and protein abundance were performed by One-way ANOVA. FACS results were analyzed using Two-way ANOVA with all statistical analysis listed performed using GraphPad Prism. Differences were considered significant at p < 0.05.

## Abbreviations

AMP: antimicrobial peptide
DEP: differentially expressed proteins (DEP)
FDR: False Discovery Rates
GO: gene ontology terms
SSDA: statistically significant differentially abundant
